# The Role of Clinical Audits in Advancing Quality and Safety in Healthcare Services: A Multiproject Analysis From a Jordanian Hospital

**DOI:** 10.7759/cureus.54764

**Published:** 2024-02-23

**Authors:** Mohammad Abu-Jeyyab, Mohammad Al-Jafari, Mostafa H El Din Moawad, Sallam Alrosan, Mohammad Al Mse'adeen

**Affiliations:** 1 School of Medicine, Mutah University, Al-Karak, JOR; 2 Internal Medicine, Al-Bashir Hospital, Amman, JOR; 3 Faculty of Medicine, Mutah University, Al-Karak, JOR; 4 Clinical Pharmacy, Faculty of Pharmacy, Alexandria University, Alexandria, EGY; 5 Internal Medicine, Saint Luke’s Health System, Kansas City, USA

**Keywords:** mixed-methods approach, adherence to guidelines, healthcare services, quality improvement, clinical audit

## Abstract

Introduction

Clinical audits have become essential instruments for evaluating and improving the standard of patient care in healthcare services. While individual clinical audits focus on particular aspects of care, multiple clinical audits across various domains, specialties, or departments provide a more comprehensive understanding of clinical practice and encourage systemic improvements.

Methodology

This study employed a mixed-methods approach to review and assess various clinical audits and quality improvement initiatives conducted at Al-Karak Governmental Hospital in southern Jordan. The study aimed to identify obstacles and possibilities of conducting clinical audits and provide suggestions for enhancing audit procedures and results. Data were collected from both retrospective and prospective sources and analyzed using descriptive and inferential statistics.

Results

The study comprised 11 audits conducted in three medical departments, namely surgery, obstetrics and gynecology (OB/GYN), and pediatrics, with a total of 618 participants. The improvements in adherence to guidelines after the second loop of all the audits were significant and showed significant improvements in adherence to guidelines, demonstrating the efficacy of clinical audits in improving clinical practice and outcomes.

Conclusions

Clinical audits are essential for maintaining and improving quality and safety in healthcare services, particularly in developing nations where emergency obstetric care is lacking. Multiple clinical audits provide a comprehensive understanding of clinical practice and encourage systemic improvements. The findings of our study suggest that clinical audits can lead to significant improvements in adherence to guidelines and better clinical outcomes. Future research should focus on identifying best practices for conducting clinical audits and evaluating their long-term viability and expandability.

## Introduction

Clinical audits serve as essential instruments in the healthcare industry for evaluating and improving the standard of patient care. They entail thorough analyses of medical procedures, protocols, and patient files to assess compliance with rules and pinpoint areas that require improvement. While individual clinical audits concentrate on particular facets of care, the idea of auditing multiple clinical audits entails carrying out a series of audits across various healthcare domains, specialties, or departments to gain a thorough understanding of overall clinical practice and encourage systemic improvements.

The quality and efficiency of healthcare delivery within an organization or across a healthcare system can be better understood by examining the results of numerous clinical audits. They make it possible to recognize patterns, trends, and variations in the way care is delivered, highlighting areas where standardization, the adoption of best practices, and performance enhancement could be done [1]. These audits offer a thorough evaluation of clinical practice by evaluating various aspects of care, including medication safety, infection control, surgical techniques, and adherence to clinical guidelines. This enables targeted interventions and practice enhancements [2].

There are many advantages to performing several clinical audits. They facilitate benchmarking against accepted standards, enable the identification and dissemination of best practices, and support the use of evidence in decision-making [3]. These audits also foster a culture of quality improvement and patient-centered care within healthcare organizations by encouraging accountability, transparency, and ongoing learning [4].

But carrying out multiple clinical audits requires careful planning, coordinating, and working with different stakeholders, including administrators, quality improvement teams, and healthcare professionals. It entails the gathering and examination of enormous amounts of data, the application of efficient auditing procedures, and the dissemination of audit findings to effect real change [5].

Allene's [6] goal was to evaluate whether the World Health Organization's (WHO) surgical safety checklist was correctly completed. According to this study, checklists can increase surgical safety, but how well they are implemented will determine how effective they are. The checklist's overall completeness during the sign-in, time-out, and sign-out periods was good [6]. The clinical audit of the WHO surgical safety checklist served as the basis for the recommendations made in this study. Checklists are used as a tool to improve team communication, teamwork, and patient safety when they are used appropriately and regularly. Additionally, the active team members should be encouraged to use the checklist regularly during their work practice to increase consistency. It is important to raise awareness, particularly among newly hired nurses and anesthesiologists. The rates of checklist compliance may also be raised by regularly auditing checklist utilization, providing refreshments on a regular basis, and providing multidisciplinary training to enhance communication. To ensure that this important tool could be used more frequently, additional training and focus on checklist use would be recommended [6].

Benjamin [7] sought to determine the viability and efficacy of utilizing a criterion-based clinical audit (CBCA) in obstetrics and gynecology (OB/GYN) departments to measure and enhance the caliber of obstetric care provided at the district hospital level in developing nations. A *before and after* design was used to audit the management of five potentially fatal obstetric complications: hemorrhage, eclampsia, genital tract infections, obstructed labor, and uterine rupture.

The purpose of this review is to provide an overview of the findings and suggestions from numerous clinical audit projects in various medical specialties that were conducted at the tertiary Al-Karak Governmental Hospital in southern Jordan. The hospital was chosen for the study because it is a typical Jordanian public healthcare institution that has completed multiple clinical audits and quality improvement initiatives in recent years.

## Materials and methods

Research design

The objective of this study was to assess the function and efficacy of clinical audits in improving the quality of healthcare services at Al-Karak Governmental Hospital, a tertiary care facility located in southern Jordan. To accomplish this objective, the study employed a mixed-methods approach, which entailed reviewing and assessing diverse clinical audits and quality improvement initiatives carried out in various medical departments of the hospital. The study aimed to identify the obstacles and possibilities of carrying out clinical audits and offer suggestions for enhancing the audit procedure and results.

Study setting

The study was conducted in Al-Karak Governmental Hospital. The hospital offers a variety of medical services, encompassing surgical procedures, OB/GYN, pediatrics, internal medicine, emergency care, and intensive care. The hospital was selected as the study's site due to its representation of a typical public healthcare facility in Jordan that has successfully executed several clinical audits and quality improvement programs in recent years.

Sample selection

The study sample comprised 618 people who participated in 11 clinical audits conducted in three medical departments of the hospital: surgery, OB/GYN, and pediatrics. The selection of these departments was based on their high audit frequency and the availability of extensive data. The audits included a range of subjects. The audits were chosen based on their pertinence to contemporary clinical practice and their conformity to national and international criteria.

Data collection

The study collected data from many sources, including both retrospective and prospective data.

Retrospective Data

The retrospective data comprised a range of papers and records about the audits, including audit reports, quality improvement plans, project evaluations, and feedback forms. The materials underwent a thorough review and analysis to enhance and validate the interview data through triangulation. Furthermore, electronic medical records and hospital databases were utilized to acquire quantifiable metrics of quality enhancement, including adherence rates to guidelines, incidence of adverse events, and patient satisfaction scores.

Prospective Data

The prospective data included semistructured interviews conducted with key stakeholders who participated in the audits, including healthcare professionals, hospital management, and patient representatives. The interviews were conducted to collect data on the goals, techniques, difficulties, and results of the audits, as well as the participants' viewpoints and encounters with the audit process and its influence on patient care. The interviews were carried out by proficient researchers utilizing a prevalidated interview guide. The interviews were documented, transcribed, and analyzed using theme analysis.

Variables

The study examined various variables that were pertinent to the research inquiries, including the attributes of the audits, such as focus area, methodology, duration, and frequency, the departments and units involved, the stakeholders and their respective roles, and the outcomes and effects of the audits, such as adherence to guidelines, quality improvement indicators, patient safety, and satisfaction.

Data analysis

The study utilized both descriptive and inferential statistics to examine and interpret the data, as outlined next.

Descriptive Analysis

The descriptive analysis entailed summarizing and presenting the data by employing suitable metrics of central tendency and dispersion. Continuous variables were represented as the median with interquartile range (IQR), whereas categorical data were reported as the count and percentage (*n*, %). Descriptive statistics were employed to depict the attributes of the audits, the participants, and the quality improvement indicators. A comparative study was conducted to evaluate the changes in adherence to recommendations over time and across different departments.

Inferential Analysis

The inferential analysis consisted of hypothesis testing and investigating the correlations between the variables using regression models. The statistical analysis was conducted using Jamovi version 2.3.16 software (The Jamovi Project, University of Sydney, Sydney, NSW, Australia). Inferential statistics were employed to assess the influence of clinical audits on adherence to recommendations and quality improvement measures while accounting for any confounding variables.

Ethical considerations

The study adhered to the ethical principles and regulations governing research with human beings. Before performing the study, ethical authorization was sought from the appropriate ethics committee. All subjects provided informed consent, guaranteeing anonymity and voluntary involvement. All personal information gathered was anonymized to guarantee privacy and adhere to data protection requirements.

Dissemination

The findings of this study were disseminated via peer-reviewed journal papers and presentations at relevant conferences, after a thorough appraisal by area specialists. The study also provided recommendations for healthcare practice and future research based on the factual information and insights gathered from the clinical audits. The study aimed to augment comprehension and use of clinical audits and quality improvement in hospital settings.

Audit cycles

The research employed a two-loop audit cycle methodology to assess the influence of clinical audits on adherence to standards. The initial audits were conducted in the first loop, using the criteria and standards set by the National Institute for Health and Care Excellence (NICE) recommendations. Analyzed data from the initial iteration was delivered to the stakeholders. After the initial loop's discoveries, modifications were implemented in clinical practice to enhance compliance with NICE standards. The adjustments encompassed routine staff training on NICE recommendations, the establishment of a uniform procedure for urine sample collection, and enhanced communication between doctors and other healthcare providers.

The second cycle involved a reassessment utilizing identical audit criteria and data-obtaining methodologies as the initial iteration. The results of the second cycle were examined and compared with those of the initial iteration to see if the modifications implemented in clinical practice had a beneficial influence on adherence to NICE guidelines. The study also evaluated the long-term viability and expandability of the modifications implemented in clinical practice.

## Results

**Figure 1:**
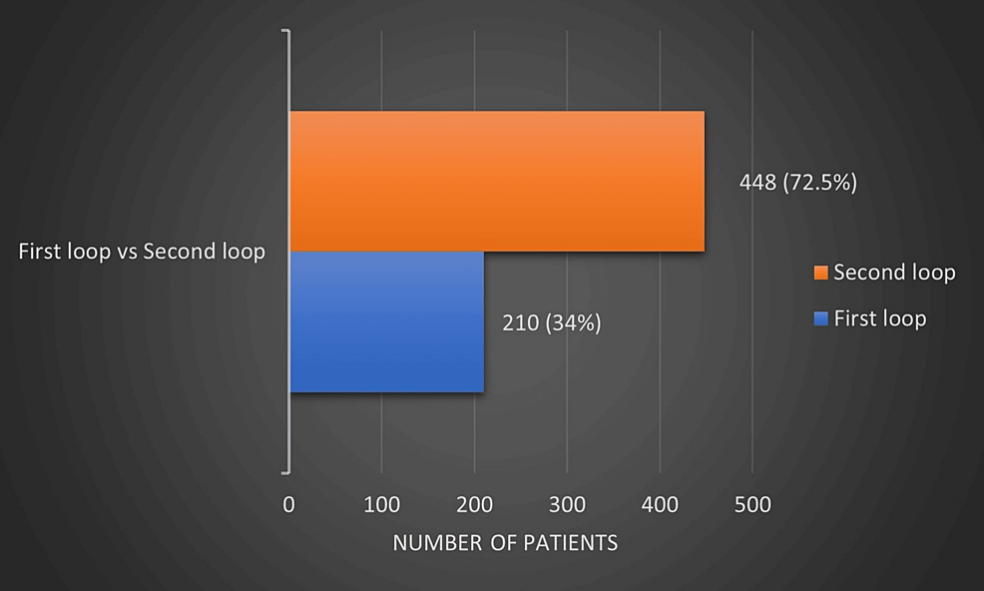
Comparison between the first loop and second loop in terms of adherence to guidelines.

The total number of participants in our projects was 618. The median age of participants was 28 with an interquartile range from 24 to 51. The projects included 370 (59.9%) females and 248 (40.1%) males. In the first loop, the total number of patients who were managed according to the guidelines was 210 (34%) (Figure 1).

After that in the second loop, the total number of patients who were managed according to the guidelines increased to 448 (72.5%) (Figure 1).

The project comprised 11 audits assessing adherence to clinical practice guidelines in Jordanian hospitals. Five were conducted in the surgery department, three in OB/GYN, and three in the pediatric department.

Regarding the audits conducted in the surgery department, the first, titled *An Audit to Reevaluate the Adherence to the Guidelines in Patients with Urinary Tract Infection at the Al-Karak Hospital in Jordan*, revealed 32 (15.2%) patients adhering to the guidelines in the first loop. In the second loop, adherence improved to 57 (12.7%) patients. The second, titled *An Audit to Evaluate the Use of eFAST in Traumatic Patients*, demonstrated that 10 (4.8%) patients adhered to guidelines, with an improvement to 37 (8.3%) patients in adherence during the second loop. The third, titled *Abdomen CT*, revealed that 20 (9.5%) patients adhered to the guidelines in the first loop, with an increase in adherence to 32 (7.2%) patients during the second loop. The fourth, titled *A Multi-centric Audit to Reevaluate the Adherence of the Guidelines in CT-KUB Imaging in Jordan*, demonstrated that 26 (12.4%) patients adhered to guidelines in the first loop, while in the second loop, adherence reached 49 (10.9%) patients. The fifth was entitled *Tranexamic acid* shown in the first loop, Tranexamic acid was not used in our department for major traumatized patients. In the second loop, adherence to the use of tranexamic acid reached 35 (7.8%) patients (Table 1).

**Table 1:** Name of audits conducted in the surgery department and their adherence to the guidelines in the first and second loops.

Name of an audit	Adherence in the first loop, *N *= 210, *n* (%)	Adherence in the second loop, *N *= 448, *n* (%)
An Audit to Reevaluate the Adherence to the Guidelines in Patients With Urinary Tract Infection at the Al-Karak Hospital in Jordan	32 (15.2%)	57 (12.7%)
An Audit to Evaluate the Use of eFAST in Traumatic Patients	10 (4.8%)	37 (8.3%)
Abdominal CT Scan in Acute Abdomen Conditions	20 (9.5%)	32 (7.2%)
A Multi-centric Audit to Reevaluate the Adherence to the Guidelines in CT-KUB Imaging in Jordan	26 (12.4%)	49 (10.9%)
Tranexamic in Trauma Patients	0 (0%)	35 (7.8%)

Regarding audits conducted in the pediatric department, the first, titled *Jaundice*, revealed that 21 (10%) patients adhered to guidelines in the first loop, while adherence reached 43 (9.6%) patients. The second, titled *Reassessment of Delivery of Healthcare Services for Mothers after Delivery to Ensure Successful Breastfeeding Outcomes*, revealed that 21 (10%) patients adhered to the guidelines. In the second loop, the adherence reached 40 (8.9%) patients. The third, titled *A Cohort Study and an Audit Project on Vitamin D Prescribing Practices for Children in Pediatric Clinics at a Tertiary Hospital in Jordan*, showed 33 (15.7%) adherence to guidelines in the first loop and 41 (9.2%) in the second loop (Table 2).

**Table 2:** Name of audits conducted in the pediatric department and their adherence to the guidelines in the first and second loops.

Name of the audit	Adherence in the first loop, *n* (%)	Adherence in the second loop, *n* (%)
Bilirubin Test for Newborns With Jaundice Risk Within the First 24 Hours	21 (10%)	43 (9.6%)
Reassessment of Delivery of Healthcare Services for Mothers After Delivery to Ensure Successful Breastfeeding Outcomes	21 (10%)	40 (8.9%)
A Cohort Study and an Audit Project on Vitamin D Prescribing Practices for Children in Pediatric Clinics at a Tertiary Hospital in Jordan	33 (15.7%)	41 (9.2%)

In audits conducted in the OB/GYN department, the first, titled *Audit To Optimize Folic Acid Prescription Before Planned Pregnancy at Al-Karak Hospital*, showed 4 (1.9%) patients adhering to guidelines in the first loop and 26 (5.8%) patients in the second loop. The second, titled *Reassessment of Blood Pressure as a Criterion for Admission and Discharge for Patients with Hypertensive Pregnancy Disorders: A Clinical Audit*, revealed adherence to guidelines in 20 patients (9.5%) during the first loop and 39 patients (8.7%) in the second loop. The third, titled *Reassessment of Practice Regarding Approach to Pain Relief During Induced Labor at a Referral Center in the South of Jordan*, showed adherence to guidelines in 23 patients (11%) during the first loop and 49 patients (10.9%) in the second loop (Table 3).

**Table 3:** Name of audits conducted in the obstetrics and gynecology (OB/GYN) department and their adherence to the guidelines in the first and second loops.

Name of an audit	Adherence in the first loop, *n* (%)	Adherence in the second loop, *n* (%)
Audit to Optimize Folic Acid Prescription Before Planned Pregnancy at Al-Karak Hospital	4 (1.9%)	26 (5.8%)
Reassessment of Blood Pressure as a Criterion for Admission and Discharge for Patients With Hypertensive Pregnancy Disorders	20 (9.5%)	39 (8.7%)
Reassessment of Practice Regarding Approach to Pain Relief During Induced Labor at a Referral Center in the South of Jordan	23 (11%)	49 (10.9%)

## Discussion

Clinical auditing is the process by which doctors and other medical professionals conduct a systematic, routine review of their clinical practice and make necessary adjustments. Reviewing professional activities through an internal audit enables evaluation of the suitability, efficacy, efficiency, and safety of the rendered services [8].

Clinical audits provide actionable recommendations for practice modification to improve clinical care and outcomes. They describe practice and ascertain whether it satisfies accepted criteria from a clinical guideline or an explicit benchmark, or not [9]. This is done by checking the practice in different departments and if this practice is aligned with the current guidelines. Then, a follow-up period is done to ensure adherence to guidelines is done. Clinical governance, which aims to ensure that patients receive the best possible care, includes clinical audits as one of its components. Further observation is conducted to confirm that improvements have been made in the delivery of healthcare after the recommendations are implemented. A clinical audit cycle is an audit that is carried out as part of a (continuous) quality improvement process and is repeated (many times, depending on requirements) [10].

In this study, five clinical audits were conducted in the surgery department, named as follows: *An Audit to Reevaluate the Adherence to the Guidelines in Patients with Urinary Tract Infection at the Al-Karak Hospital in Jordan*, *An Audit to Evaluate the Use of eFAST in Traumatic Patients*, *Abdomen CT*, *A Multi-centric Audit to Reevaluate the Adherence of the Guidelines in CT-KUB Imaging in Jordan*, and *Tranexamic Acid*. The second loop of all five audits showed significant improvement in adherence to the guidelines compared to the first loop. We carried out three audits in the pediatric department, the first was titled *Jaundice*, the second was titled *Reassessment of Delivery of Healthcare Services for Mothers after Delivery to Ensure Successful Breastfeeding Outcomes*, and the third was titled *A Cohort Study and an Audit Project on Vitamin D Prescribing Practices for Children in Pediatric Clinics at a Tertiary Hospital in Jordan*. These also showed improvement in the sticking to guidelines recommendation in the second loop compared to the first one. The third was the OB/GYN department with three clinical audits, the first was titled *Audit to Optimize Folic Acid Prescription Before Planned Pregnancy at Al-Karak Hospital*, the second was titled *Reassessment of Blood Pressure as a Criterion for Admission and Discharge for Patients With Hypertensive Pregnancy Disorders*, and the third was entitled *Reassessment of Practice Regarding Approach to Pain Relief During Induced Labor at a Referral Center in the South of Jordan*. These audits also demonstrated improvement in adherence to guidelines after the second loop.

Surgical audits have gained importance in today's surgical practice and are now essential for surgeons to undertake, contributing to their ongoing professional development and dedication. This commitment, in turn, leads to better practice habits. In developed nations, there exists a highly effective national audit and comparative audit services system [11]. Alqudah et al. [12] investigated the quality of surgical audits in a tertiary hospital in Jordan. This study showed that all elements of surgical audits had improved from baseline and that electronic materials and better personnel education could achieve higher performance in the surgical department. A study by Bindroo and Saraf [11] aimed to assess the surgical audit with its associated morbidity and mortality. They concluded that this audit is very important to implement protective actions in a way that decreases mortality rates and increases good outcomes of surgery.

To apply the clinical audit process to the treatment of obesity and overweight children, Limauro et al. [6] conducted a study. Through an examination of their clinical procedures, they modified the feeding and weaning protocol for up to 36 months, primarily minimizing excess protein and sugar. This modification affected the post-audit group of toddlers born in 2010, 2011, and 2012. A shift in pediatricians' dietary approaches resulted in a decrease in the prevalence of overweight/obesity in children aged 24 to 36 months, from 26.3% in the pre-audit group to 13.9% in the post-audit group (*P* < 0.0001). This study found that the clinical audit is a useful tool for finding errors in medical procedures and assisting in attitude modification when needed, both of which have a positive impact on the standard of care [8].

Clinical audits prompted pediatricians to update their nutritional counseling protocols and implement pertinent changes related to dietary intake, food literacy assessment, and parent misconceptions about breastfeeding infants. More specifically, it was estimated that between 13 and 36 months of age, a child's protein intake would decrease from more than 3.5 g/kg to roughly 2 g/kg as a result of changes in pediatricians' practices regarding the selection and amount of high-protein foods [13].

This shows that applying clinical audits as in our study will be associated with better improvement in children's conditions such as nutritional conditions, jaundice, vitamin D supplementation, and care after delivery. Pediatricians benefit a lot from clinical audits, and this turns into better health and life for children.

More than 500,000 women worldwide pass away from pregnancy- and childbirth-related causes every year; developing nations account for more than 99% of these deaths [7]. Poor emergency obstetric care at the first referral level is one of the main causes of the high rate of maternal deaths in the developing world. Both developed and developing countries share similar basic principles for the optimal management of obstetric complications that pose a life-threatening risk. Although it has not yet been widely implemented in developing nations, clinical audits are currently regularly employed and acknowledged as a component of quality assurance in the health services of many developed countries [14].

To implement CBCA, clinicians must first agree upon a set of succinct standards for high-quality care, taking into account the available resources [15]. Audit assistants who are not medically qualified examine pertinent patients' medical records and gather information to evaluate if the care was provided in accordance with established standards. When and where indicated, the audit team and hospital employees collaboratively determine which practices to modify and subsequently implement those changes. The fact that the very act of disclosing that a predetermined standard of care is not being met - or, as one participating hospital midwife put it, "by holding up a mirror to ourselves" - also pinpoints the precise adjustments that must be made in clinical practice is one of the unique characteristics of CBCA. The percentage of cases where management satisfied the established standards for high-quality care is changed to determine how effective this auditing method was [7].

Clinical audits generally have the drawback of not being able to show causal relationships with clinical outcomes, unlike randomized clinical trials, even though they can show changes in care quality process items. This highlights the importance of aligning audits with real clinical trials and adhering to guidelines. According to a systematic review, audits typically only produce modest practice improvements, and their efficacy depends on both baseline performance and how feedback is given [16]. We advise providing ongoing auditing in conjunction with first-rate assistance and monetary rewards to encourage bigger advancements.

## Conclusions

In conclusion, this review of multiple clinical audit projects conducted at a tertiary care facility in Southern Jordan highlights the positive impact of clinical audits in improving the quality and safety of healthcare services. The findings demonstrate significant improvements in adherence to guidelines across various specialties, with notable enhancements in the OB/GYN department. Clinical audits play a crucial role in promoting a culture of quality improvement and patient-centered care, providing actionable recommendations for practice modification and supporting evidence-based decision-making.

The study emphasizes the importance of ongoing auditing, support, and incentives to encourage larger improvements in healthcare services. Furthermore, clinical audits are particularly vital in developing nations where emergency obstetric care is lacking, making them essential for maintaining and improving healthcare quality and safety.

Future research should focus on identifying best practices for conducting clinical audits, evaluating their long-term viability, and expanding their implementation to other healthcare facilities. By utilizing clinical audits as a continuous quality improvement tool, healthcare organizations can ensure they are delivering optimal patient care and driving positive outcomes.

## References

[1] Braithwaite J, Runciman WB, Merry AF (2009). Towards safer, better healthcare: harnessing the natural properties of complex sociotechnical systems. Qual Saf Health Care.

[2] National Institute for Clinical Excellence (2010). Principles for Best Practice in Clinical Audit. Principles for Best Practice in
Clinical Audit.

[3] (2024). Audit and Service Improvement. https://www.nice.org.uk/about/what-we-do/into-practice/audit-and-service-improvement.

[4] Royal College of Physicians. Clinical Audit and Quality Improvement. . (2019 (2019). Royal College of Physicians. Clinical Audit and Quality Improvement. https://www.rcplondon.ac.uk/projects/outputs/clinical-audit-and-quality-improvement.

[5] (2011). National Health Service. Clinical Audit Policy. https://www.uhbristol.nhs.uk/media/3563138/clinicalauditpolicy-4_1.pdf.

[6] Limauro R, Gallo P, Cioffi L, Antignani A, Cioffi V, Calella P, Valerio G (2020). Clinical audit in the pediatric primary care office and overweight prevention in toddlers. BMC Pediatr.

[7] Benjamin A (2008). Audit: how to do it in practice. BMJ.

[8] Skull S (2020). Embedding clinical audit into everyday practice: essential methodology for all clinicians. J Paediatr Child Health.

[9] Bilal A, Salim M, Muslim M (2005). Two years audit of thoracic surgery department at Peshawar. Pak J Med Sci.

[10] Allene MD (2020). Clinical audit on World Health Organization surgical safety checklist completion at Debre Berhan comprehensive specialized hospital: a prospective cohort study. Int J Surg Open.

[11] Bindroo S, Saraf R (2015). Surgical mortality audit-lessons learned in a developing nation. Int Surg.

[12] Alqudah M, Aloqaily M, Rabadi A (2022). The value of auditing surgical records in a tertiary hospital setting. Cureus.

[13] Hörnell A, Lagström H, Lande B, Thorsdottir I (2013). Protein intake from 0 to 18 years of age and its relation to health: a systematic literature review for the 5th Nordic Nutrition Recommendations. Food Nutr Res.

[14] Geleto A, Chojenta C, Musa A, Loxton D (2018). Barriers to access and utilization of emergency obstetric care at health facilities in sub-Saharan Africa: a systematic review of literature. Syst Rev.

[15] Amado BG, Arce R, Fariña F, Vilariño M (2016). Criteria-based content analysis (CBCA) reality criteria in adults: a meta-analytic review. Int J Clin Health Psychol.

[16] Ivers N, Jamtvedt G, Flottorp S (2012). Audit and feedback: effects on professional practice and healthcare outcomes. Cochrane Database Syst Rev.

